# Unfair Trade e-Waste in Africa

**DOI:** 10.1289/ehp.114-a232

**Published:** 2006-04

**Authors:** Charles W. Schmidt

The bright and dark sides of Africa’s information technology sector are both evident at the Ikeja Computer Village, near Lagos, Nigeria. Thousands of vendors pack this bustling market, one of three major hubs where imported used electronics are repaired and sold. Computers, fax machines, cell phones—if you want one, you can find it here, spruced up and ready to buy. But beyond the thriving storefronts and the piles of refurbished wares, a darker picture emerges. Up to 75% of the electronics shipped to the Computer Village are irreparable junk, according to the Computer and Allied Product Dealers Association of Nigeria, a local industry group. Nigeria has a thriving repair market, but no capacity to safely deal with electronic waste, most of which winds up in landfills and informal dumps. That’s a problem, because this “e-waste” can be toxic: much of it is loaded with potentially toxic metals including lead, cadmium, and mercury. What’s more, electronic components are usually housed in plastic casings that spew carcinogenic dioxins and polyaromatic hydrocarbons when burned.

## Emerging Dumping Grounds

Hungry for information technology but with a limited capacity to manufacture it, Africa has become the world’s latest destination for obsolete electronic equipment. Much of this material is more or less functional and provided in good faith by well-meaning donors. But the brokers who arrange these exports often pad shipping containers with useless junk, essentially saddling African importers with electronic garbage. In 2002, the Basel Action Network (BAN), a Seattle-based environmental group, made headlines with its investigation of e-waste exports to Asia [see “e-Junk Explosion,” *EHP* 110:A188–A194 (2002)]. More recently, BAN explored Africa’s e-waste problem, and described its findings in an October 2005 report titled *The Digital Dump: Exporting Re-use and Abuse to Africa*.

BAN coordinator Jim Puckett, who visited Nigeria as part of that investigation, saw enormous piles of e-waste throughout the countryside, much of it routed through Lagos, Africa’s largest port. “We saw people using e-waste to fill in swamps,” Puckett recalls. “Whenever the piles got too high, they would torch them. . . . Residents complained about breathing the fumes, but the dumps were never cleaned up. We saw kids roaming barefoot over this material, not to mention chickens and goats [which wind up in the local diet].”

Puckett says the dumps near Lagos could be the tip of an iceberg. No one knows for sure because there are virtually no data concerning the global e-waste trade—harmonized tariff schedules that dictate fees for export commodities don’t assign codes to waste electronics other than batteries. There are tariff classifications for scrap (e.g., plastic, metal) and for new electronics by type (e.g., computer monitors, TV sets). Because the importers don’t want to pay tariffs on a five-year-old computer based on the price of a new one, they often use scrap classifications, measured in pounds, says Robin Ingenthron, acting president of the World Reuse, Repair and Recycling Association (WR3A), a nonprofit group trying to establish fair trade standards for the practice. Consequently, the volume, characteristics, and destinations of e-waste exports are shrouded in mystery.

BAN’s investigation—among the first of its kind in Africa—was limited to areas near Lagos, followed by a week-long foray into neighboring Niger, a landlocked country. Based on BAN’s firsthand observations and other anecdotal reports, Puckett now believes e-wastes are passing through African port cities that, in addition to Lagos, include Mombasa, Dar es Salaam, and Cairo. Puckett didn’t encounter e-waste in Niger and speculates that this is at least in part because the inland country has no port.

An estimated 500 shipping containers loaded with secondhand electronic equipment pass through Lagos each month, BAN’s investigation found. Each container can be packed, on average, with a load equal in volume to 800 computer monitors or central processing units (CPUs), or 350 large TV sets. Local experts cited by BAN estimate that anywhere from 25% to 75% of this material is useless. Assuming the low end of this range, one could hypothesize that volumes of e-waste equal to 100,000 computers or CPUs, or 44,000 TV sets, enter Africa each month through Lagos alone.

## The E-Waste Trade

Why do African importers pay for electronic junk they can’t sell? If the contents of shipping containers are purchased by weight, not by the combined value of what’s inside them, then waste can be transported by “averaging” the load. It costs an average of US$5,000 to ship a 40-foot container full of used electronics from the United States to Africa, according to Jim Lynch, senior program manager for computer recycling and reuse at Compu-Mentor, a San Francisco–based nonprofit organization. Once there, some of this equipment can fetch a high price: Olayemi Adesanya, BAN’s logistical coordinator in Nigeria, says a functional Pentium III computer sells for about US$130 on Nigerian markets, while a working 27-inch TV might sell for US$50. (Scrap components—especially working hard drives—can also be readily sold in Nigeria to supply an emerging reassembly industry.) Therefore, it doesn’t take many working units to cover shipping costs. Indeed only 40 good Pentium III computers pays for an entire container, leaving a comfortable margin for profit even if the container is loaded with mostly unusable waste.

The question of who’s selling e-waste to Africa is harder to answer. Used electronics travel murky routes populated by numerous recyclers and brokers working in an unregulated market, devoid of government certification programs. Electronics recyclers are at the top of the supply chain. These companies incur tremendous overhead expenses—to recycle a single monitor in the United States, for instance, can cost up to $15.

Many recyclers run legitimate operations that absorb these costs and profit from refurbished equipment sales and fees charged for accepting old, unsalable material. But others are not so scrupulous. According to one anonymous recycler, it’s not uncommon for companies to coordinate with exporters to ship junk overseas. In some cases, exporters negotiate with buyers in developing countries, who dictate the amount of junk they will accept in exchange for a specified number of high-value items. “I could come up with half a load of good stuff and say, ‘If you want it, you have to take the bad,’ and sell it all by the pound,” the recycler says. “Then the guy in Africa will crunch the numbers and say, ‘OK, if you put a few more Pentium IIIs in there, you’ve got a deal.’”

In other cases, the recycler adds, the deals are less defined—exporters simply load containers with junk, and sell it by the pound to inexperienced buyers who don’t know to negotiate content from the outset. These cases are rare, however, and buyers stuck with containers full of worthless junk aren’t likely to make the same mistake again, he says.

By the same token, says Ingenthron, some inexperienced exporters might unwittingly send a Cisco router worth $15,000 in a container load of “mixed electronics.” The WR3A refers to loads like those as “lottery tickets.”

Ingenthron stresses that not all waste exports are bad. Asian importers, for instance, can sell working cathode ray tubes (CRTs), which contain up to four pounds of lead each, to electronics manufacturers who use them to make new products. Other importers may purchase broken CRT glass to be manufactured into new CRTs. “If you have containers full of cleaned, processed broken CRT glass going to Asian CRT furnaces, that’s good for the environment,” he says. “Otherwise, you have to mine for the metals.”

Asia does, in fact, have a thriving electronics recovery industry that supplies manufacturers with recycled raw materials. While the practice does have its benefits, as noted above, it also exploits women and child laborers who cook circuit boards, burn cables, and submerge equipment in toxic acids to extract precious metals such as copper. BAN documented these practices, which have dire health and ecological consequences, during its 2002 and 2004 visits to China. However, BAN investigators didn’t witness this type of activity in Nigeria. Puckett speculates this might be because waste volumes there aren’t yet high enough to realize profits from recovery. In that case, he suggests, it could be just a matter of time before the same hazardous e-waste extraction methods observed in China emerge in the Lagos street economy.

## Stemming the Tide

Numerous efforts to limit the flow of e-waste to developing countries are under way even as export volumes continue to grow. For its part, BAN has pushed for U.S. ratification of the Basel Convention, an international treaty drafted in 1989 that aims to prevent hazardous wastes from being dumped in the developing world (wastes exported for reuse and recycling are allowed under the treaty, however). The United States is one of the few countries in the world that have not yet ratified the convention. As it stands now, e-waste exports from the United States are illegal only under the Resource Conservation and Recovery Act, and within that law, only if the exports wind up disposed of overseas. As long as the export goal is “recycling,” U.S. shippers can legally send e-wastes wherever they wish.

Despite repeated inquiries, the E P A would not elaborate further on the U.S. position regarding e-waste exports and their associated environmental impacts, except to say the agency has for several years negotiated with the Organisation for Economic Co-operation and Development on a program that will provide “greater assurance that exports of recyclable materials will be managed in an environmentally sound manner.” No release date for the program was provided.

Meanwhile, a number of voluntary e-waste export reduction efforts are under way in the United States. In 2003, the EPA created the “Plug-In To eCycling” program, which promotes safe domestic recycling of electronic equipment by consumers and businesses. BAN has produced a document it calls the “Electronic Recycler’s Pledge of True Stewardship,” which can be signed by companies that promise not to send e-wastes to landfills, incinerators, or developing countries. And the WR3A has developed a new “e-certification program” to help e-waste generators find recyclers who can process their deliveries in an environmentally sustainable way.

All these programs have their work cut out for them—the electronics industry thrives on obsolescence. Computers, cell phones, and other gadgets go out of date quickly, sometimes within months of release. Indeed, e-waste is now considered the fastest growing segment of the municipal waste stream in the United States. But the United States is also weak in legitimate repair and reuse, discarding items that represent real income for educated repairpeople in other countries. And Africa, with its own economy dependent on the leftovers, is left picking through electronic trash. “There’s just a lot more junk going to Africa now,” Ingenthron says. “In Asia, the buyers tend to know more about the material than the sellers. But in Africa, it’s the other way around.”

## Figures and Tables

**Figure:**
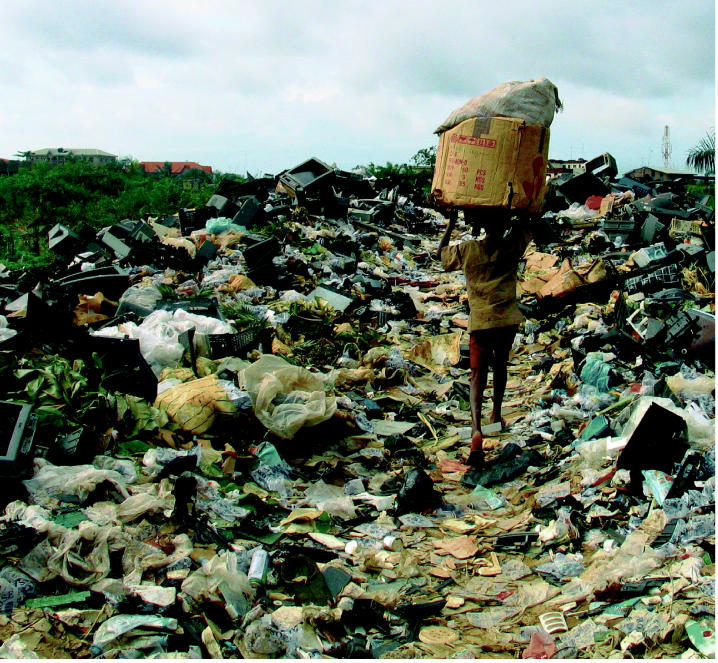
Bad reception. A boy hauls electronic waste from the Alaba Market in Lagos, Nigeria, to a nearby informal dump sitting atop a swamp. Imported televisions and computers that cannot be repaired get deposited here, and later burned.

**Figure:**
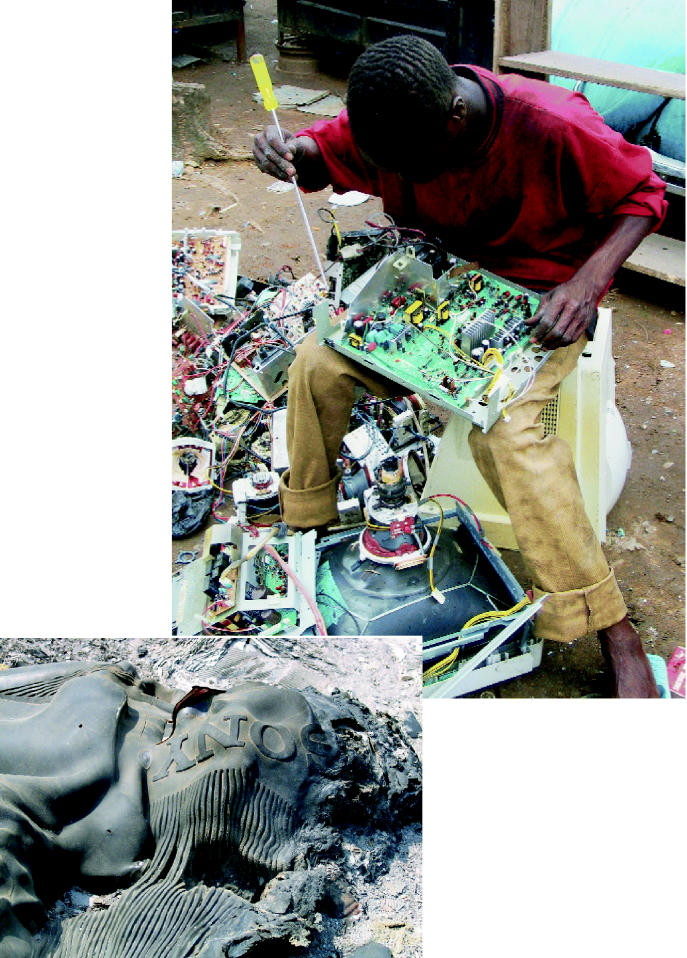
Lots of trash, very little treasure. (above) Thousands of Nigerians are involved in repairing and reselling imported used electronic equipment. Unfortunately, much of the imported electronics cannot be repaired and are instead dumped and burned. (left) Brominated flame retardants and heavy metals in plastics can yield toxic emissions when casings are incinerated.

